# Experiences of dyslexia in GP training in the UK: a qualitative study

**DOI:** 10.3399/BJGPO.2025.0121

**Published:** 2026-06-03

**Authors:** Suhail Amin Tarafdar, Kalman Winston, Noha Seoudi, Deborah Lowry, Ruoyin Luo

**Affiliations:** 1 College of Medicine and Dentistry (CoMD), Outreach Centre of Ulster University, Birmingham, UK; 2 School of Pharmacy and Pharmaceutical Sciences, Ulster University, Coleraine, UK; 3 Institute of Continuing Education, University of Cambridge, Cambridge, UK; 4 Warwick Medical School, Warwick University, Coventry, UK; 5 NHS England Workforce, Training and Education Directorate, West Midlands, Birmingham, UK; 6 Department of Public Health and Primary Care, University of Cambridge, Cambridge, UK; 7 Institute of Continuing Education, University of Cambridge, Cambridge, UK; 8 Institute of Dentistry, Queen Mary University of London, London, UK; 9 Barts Health NHS Trust, Barts Health Dental Hospital, London, UK; 10 School of Pharmacy and Pharmaceutical Sciences, Ulster University, Coleraine, UK

**Keywords:** postgraduate education, learning disabilities, qualitative research, dyslexia

## Abstract

**Background:**

Dyslexia is a neurodevelopmental learning difficulty that affects the acquisition of reading and spelling. Dyslexic medical residents can experience difficulties with assessments and workload, although reasonable adjustments are helpful. Nonetheless, there is limited evidence regarding the experiences of dyslexia within family medicine and general practice training.

**Aim:**

To explore experiences of dyslexia in GP training in the UK, and identify adaptive strategies to improve training experiences.

**Design & setting:**

This was a qualitative study comprising detailed semi-structured, one-to-one interviews. Participants were dyslexic GPs or residents, or educators of dyslexic GP residents, within UK training programmes.

**Method:**

Interviews were conducted online, recorded, and transcribed verbatim. Thematic analysis was undertaken of the data.

**Results:**

There were 26 participants with five themes identified. Getting support could be protracted, owing to lack of awareness, stigma, and attitudes. Furthermore, there were different challenges faced by dyslexic doctors within hospital medicine and general practice settings. Dyslexia also informed the career progression of dyslexic doctors, including after completion of GP training. Moreover, adaptive strategies could enhance performance in GP training assessments. Likewise, workplace adjustments could improve the experiences of dyslexic doctors in general practice.

**Conclusion:**

Further training regarding dyslexia is required for GP residents and educators. Programmes should foster a positive, inclusive culture. Adaptive strategies can be employed within primary care to improve experiences. Moreover, creating a toolkit for dyslexia in GP training can be beneficial for residents and educators, with further research also warranted to explore dyslexia among international medical graduates (IMGs).

## How this fits in

There is a paucity of research exploring dyslexia in GP training and following completion of specialty training. This study has identified the need for greater awareness of dyslexia for GP residents and educators, in addition to incorporating an inclusive, supportive environment. There are specific differences in the experience of dyslexic doctors within general practice and hospital environments, which reflects the adaptive strategies identified with primary care settings. A GP-specific toolkit could improve knowledge and understanding of dyslexia, while further research is needed to explore international medical graduates’ experiences of dyslexia, and the impact of recent changes to Royal College of General Practitioners assessments.

## Introduction

Dyslexia is a neurodevelopmental learning difficulty that affects the acquisition of reading and spelling. It can also affect information processing, working memory, and orthographic skills.^1,2^ Dyslexia should be considered within a wider neurodiversity approach, whereby diversity is expected rather than all individuals conforming to neurotypicality, with disability arising from the interaction between neurodiverse individuals and an inconducive neurotypical environment. To address this, society should value diversity and remodel environments to make them conducive for people with dyslexia, while dyslexic individuals can implement adaptive strategies.^3^ The authors recognise differences in individual preferences for identity-first language ("dyslexic doctors") versus person-first language ("doctors with dyslexia"); the authors have chosen identity-first language for this article, while acknowledging that some readers may feel less comfortable with this choice.

The UK prevalence of dyslexia is 10%.^4^ It represents 80% of all learning difficulties,^5^ with 4.6% of UK medical students declaring a specific learning difficulty (SpLD) in 2018.^6^ Nonetheless, this may be an underestimation, owing to non-disclosure.^7,8^ Dyslexia is associated with stigma, negative attitudes, workplace difficulties, and assessment challenges.^7–10^ However, adaptive strategies can reduce burden.^8^ Moreover, provision of reasonable adjustments is a legal requirement under many jurisdictions, including the UK Equality Act 2010 and the European Union Charter of Fundamental Rights.^11,12^

GP training within the UK lasts 3 years, with the competency-based curriculum comprising non-clinical domains and clinical and life-stage topics, and incorporating a range of formative, summative and workplace-based assessments (WPBAs).^13^ GP residents (‘registrars’) in the UK must complete three assessment components: the written applied knowledge test (AKT), the simulated consultation assessment (SCA), and WPBAs, to achieve their certificate of completion of training (CCT).^14^

One recent systematic review found that there is limited evidence on experiences of dyslexia within GP training and following completion of specialty training.^8^ UK studies indicate that residents with SpLD are more likely to experience multiple exam attempts and failure during GP training, and that reasonable adjustments are effective for the AKT.^15–18^ They are also more likely to experience difficulties with WPBAs and adverse outcomes at their annual progression review.^15,18^ One study of UK GP residents, including dyslexic participants, found that personal, professional, and social factors were associated with failure to progress.^19^ A more recent qualitative study of SpLD in UK GP training identified that early detection and appreciating the intersectionality between neurodiversity and being an international medical graduate (IMG) are important.^20^ It outlined the need for learning strategies, awareness, psychological support, and equipment to assist during training.^20^ The aim of this qualitative study was to explore experiences of dyslexia in GP training in the UK, and identify adaptive strategies to improve dyslexic residents’ experiences.

## Method

### Research design

As this study explored lived experiences and strategies, a phenomenological qualitative approach was selected.^21^

### Recruitment

Participants were dyslexic GPs who completed UK training, or dyslexic residents undertaking UK GP training. Educators of dyslexic GP residents within UK training were also included.

Recruitment was through email invitation from the GP school to all resident doctors and educators within the West Midlands. Snowball sampling enhanced recruitment, where participants disseminated the invitation beyond the West Midlands.^22^

### Data collection

All participants underwent individual, semi-structured interviews via Microsoft Teams, between June and September 2024, allowing in-depth data to be collected.^21,23^ Consent was obtained and interviews undertaken using a topic guide (supplementary file 2), based on a prior systematic review.^8^ Interview questions were open-ended to allow participants to elucidate lived experiences in their own words. Furthermore, ideas that emerged during data collection were explored in subsequent interviews.^21,23^ The number of participants was based on pragmatism and achievement of data adequacy:^24,25^ towards the end of the data collection, no further themes were identified; there was representation from dyslexic resident and educator participants; and rich data sufficient to answer the aims of the study had been collected.^24–26^ Interviews were recorded and transcribed verbatim, with all participant-identifiable information anonymised.

### Data analysis

Inductive thematic analysis of all transcripts was undertaken, involving six steps: data familiarisation; line-by-line analysis to generate initial codes; searching for themes; reviewing themes and their relationships; defining and naming themes, ensuring that they captured the essence of the participants’ lived experiences; and producing a report. Coding was manually undertaken to enhance familiarity and immersion with the data.^21,27^

To improve trustworthiness, detailed participant characteristics were collected, in addition to member checking after each interview. A reflexive diary was logged by the lead author (ST). While the lead undertook all interviews and analysis, a proportion of transcripts were also analysed by two other researchers.^28–30^ Recommendations from the consolidated criteria for reporting qualitative research (COREQ) checklist were considered for designing and reporting the study (supplementary table 1).^31^

## Results

### Participant characteristics

Table 1 outlines participant characteristics. There were 26 participants: 20 completed their primary medical qualification in the UK, while six were IMGs.

Thirteen participants were dyslexic, with six current GP residents: five in their third year of training (GPST3) and one in their first year (GPST1). The remaining seven dyslexic participants had completed GP training.

**Table 1.** A table outlining the characteristics of participants included within the study

Participant number	Sex	Age in years	Ethnicity	Stage of training	PMQ: UK or IMG	Year completed PMQ	Year completed GP training	Educator of GP residents with dyslexia, year started, role(s)	Dyslexia, year and location diagnosed
**1**	Female	49	Asian	CCT	UK	2011	2020	No	Yes, 2005, medical school
**2**	Female	44	White	CCT	UK	2007	2012	Yes: N/A, ES	Yes, 2012, GPST3
**3**	Female	34	White	CCT	UK	2013	2021	No	Yes, 2021, GPST3
**4**	Male	38	Asian	CCT	IMG	2012	2022	No	Yes, 2020, GPST3
**5**	Female	44	White	CCT	UK	2003	2008	No	Yes, 2018, post-GP training
**6**	Female	35	White	CCT	UK	2018	2023	No	Yes, 2011, non-medical degree
**7**	Male	31	Asian	GPST3	UK	2018	N/A	No	Yes, 2022, GPST1
**8**	Female	35	White	GPST3	UK	2017	N/A	No	Yes, 2007, non-medical degree
**9**	Female	53	White	GPST3	IMG	1995	N/A	No	Yes, 2024, GPST2
**10**	Female	42	Asian	CCT	UK	2006	2011	Yes, 2013, ES and TPD	Yes, 2013, post-GP training
**11**	Male	28	Asian	GPST1	UK	2020	N/A	No	Yes, 2015, medical degree
**12**	Male	37	Asian	GPST3	IMG	2012	N/A	No	Yes, 2023, GPST3
**13**	Male	36	Arab	GPST3	IMG	2014	N/A	No	Yes, 2023, GPST2
**14**	Female	53	Asian	CCT	IMG	1993	2006	Yes, 2010, ES and TPD	No
**15**	Female	45	White	CCT	UK	2002	2008	Yes, 2008, ES and TPD	No
**16**	Male	38	Asian	CCT	UK	2009	2015	Yes, 2018, ES and TPD	No
**17**	Female	58	White	CCT	UK	1989	1995	Yes, 2008, ES	No
**18**	Male	49	Asian	CCT	IMG	1992	2021	Yes, 2023, ES and TPD	No
**19**	Female	36	White	CCT	UK	2011	2023	Yes, 2023, TPD	No
**20**	Male	34	White	CCT	UK	2012	2017	Yes, 2020, ES and TPD	No
**21**	Male	56	White	CCT	UK	1992	1996	Yes, 2009, ES and TPD	No
**22**	Male	63	Asian	CCT	UK	1985	1990	Yes, 1992, ES and TPD	No
**23**	Male	41	Asian	CCT	UK	2006	2011	Yes, 2013, ES and TPD	No
**24**	Female	49	Asian	CCT	UK	1998	2002	Yes, 2005, ES and TPD	No
**25**	Male	64	Asian	CCT	UK	1983	1988	Yes, 2000, ES and TPD	No
**26**	Male	54	Asian	CCT	UK	1992	1997	Yes, 2004, ES, TPD and AD	No

AD = associate dean. CCT = certificate of completion of training (that is, completed GP training). ES = educational supervisor. GPST = general practice specialty training. PMQ = primary medical qualification. N/A = not applicable. TPD = training programme director

Fifteen participants were educators, two of whom were also dyslexic. All educators had completed GP training and most had experienced multiple roles: 13 were educational supervisors, 13 training programme directors, and one an associate dean.

### Findings from thematic analysis

There were five overarching themes, divided into sub-themes. The relationship between themes and sub-themes is summarised in the thematic map within Figure 1.

**Figure 1.**
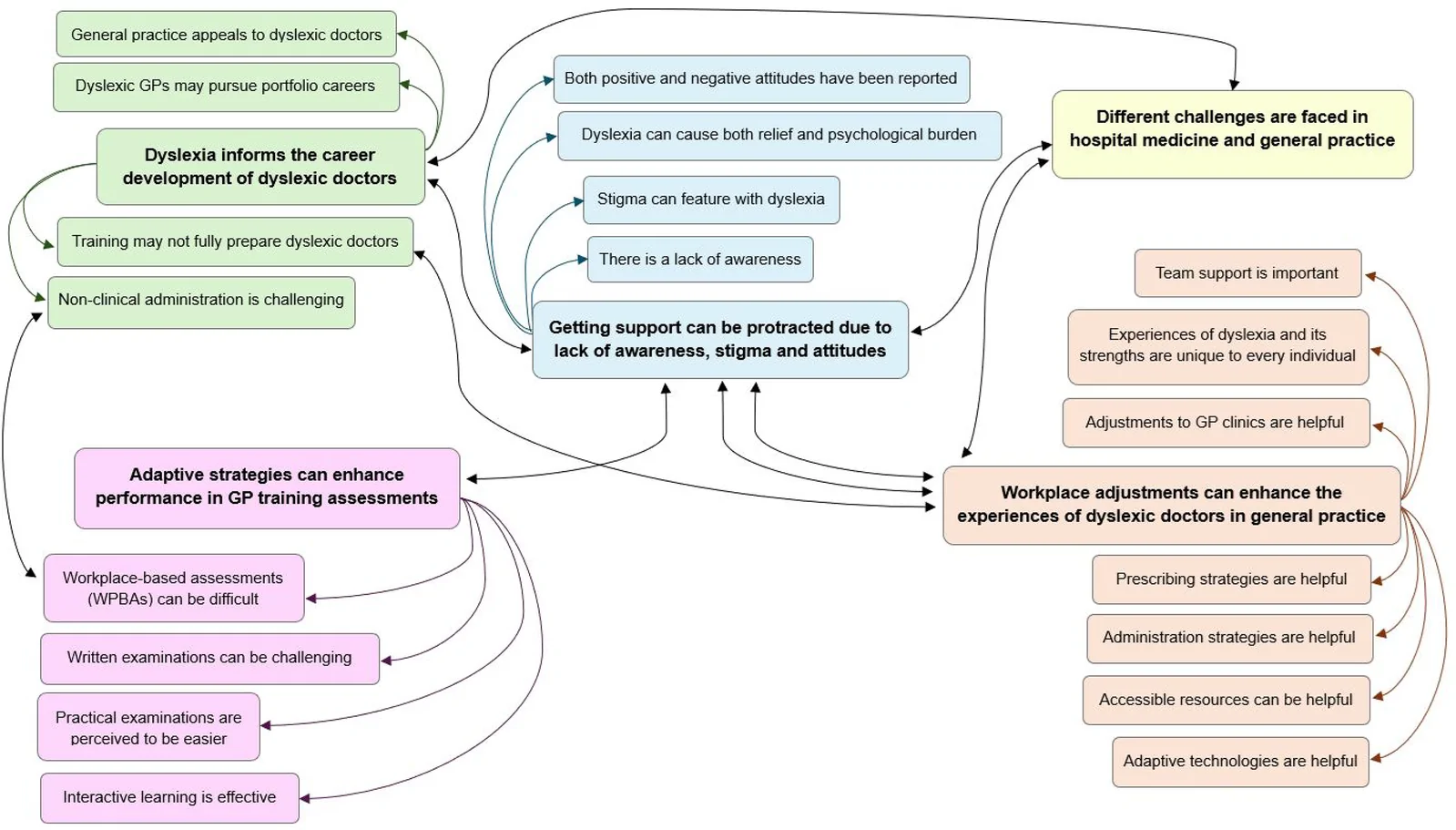
Thematic map outlining relationship between themes (in bold) and sub-themes

The themes in Table 2 were broadly identified across all participants, although some themes were emphasised within a particular group.

**Table 2.** Table highlighting themes that were emphasised in a particular group of participants from the thematic analysis

Experiences of dyslexic GP residents	Experiences of educators	Experiences post-CCT dyslexic GPs
Lack of awareness and support Difficulties with assessments: AKT, SCA, WPBAs Difficulties within the workplace related to time-keeping and processing Challenges within hospital medicine rotations	Challenges heightened at the intersection of dyslexia with IMG doctors Need for more awareness and training Concerns related to screening training doctors for dyslexia Concerns regarding feasibility of some adjustments that raise issues of fairness WPBAs especially challenging, particularly portfolio engagement	Ongoing difficulties with time-keeping Difficulties with negotiating reasonable adjustments post-CCT Benefits of pursuing portfolio careers Challenges with non-clinical administration post-CCT, especially appraisal and revalidation

AKT = applied knowledge test. CCT = certificate of completion of training. IMG = international medical graduate. SCA = simulated consultation assessment. WPBA = workplace-based assessments

Getting support can be protracted owing to lack of awareness, stigma, and attitudes

#### There is a lack of awareness

Dyslexia was often diagnosed late, owing to poor awareness and masking. Educators highlighted a noticeable rise in diagnosis within IMGs, perceived to be following an increase in IMG residents and lack of screening in their primary medical qualification universities. Furthermore, some educators wondered *‘whether there’s a second language dyslexia sort of thing*’ (P21).

While participants did feel that awareness was improving, knowledge regarding its features and adaptive strategies, was lacking:

*‘There’s a lot of feeling that it’s all to do with spelling and that’s not necessarily a full representation*.’ (P3)

#### Stigma can feature with dyslexia

While people were *‘moving away*’ (P2) from stigma, it still featured, particularly among IMGs: *‘There’s a stigma. When I referred the overseas trainee there was lots of hesitation*’ (P14).

Several participants disclosed their diagnosis to access support; some even comfortably informed patients. Nonetheless, others expressed reluctance:

*‘If I was to tell them that I had dyslexia, it would mean I'm not as quick. I'm not as good. I just didn't feel comfortable saying that*.’ (P6)

#### Both positive and negative attitudes have been reported

Participants reported negative attitudes from colleagues and supervisors including accusations of being unfairly *‘facilitated for no reason*’ (P12), discrimination, and lack of support:

*‘Trainers are just not engaged or very blasé towards dyslexia*.’ (P11)

Nonetheless, positive attitudes were also reported from colleagues and educators, including professional support and wellbeing (PSW), where provision of coaching was helpful. Educators expressed willingness to learn and adapt to the needs of dyslexic residents, with close relationships formed: *‘You end up more bonded when you've had to work harder to get people through*’ (P22).

#### Dyslexia can cause both relief and psychological burden

Psychological burden was high among dyslexic GPs and residents, including burnout, stress, and depression:

*‘There’s a risk of burnout during training and post-training. I've probably burnt out three, four times*.’ (P1)

Following diagnosis, participants described a *‘grief process*’ (P5), although others described *‘relief*’ (P3), as it explained their struggles. Some made positive adjustments following diagnosis: *‘I started to relax, became more self-aware and nicer to myself*’ (P11).

### Different challenges are faced in hospital medicine and general practice

Participants highlighted differences in the challenges of dyslexia between hospital medicine and general practice. Within hospitals, being on-call, holding a bleep to answer medical calls, and caring for sick patients were *‘stressful with dyslexia*’ (P8). Other challenges included ward rounds; handovers; and writing or reading notes and discharge summaries.

Nonetheless, given larger teams within hospitals, tasks could be distributed according to workload and individual strengths. In contrast, the autonomous nature of general practice, comprising a smaller team, made this challenging:

*‘In the hospital, you can be adjusted within your team. In general practice, you’re doing everything. You’re doing the prescription, the results, seeing the patient. So that is a more stressful experience*.’ (P12)

Participants felt that difficulties manifested within general practice owing to processing and time-keeping issues associated with shorter appointment lengths, the large volume of patients and tasks, and a lack of handover.

Because practices were smaller and used electronic records, *‘in GP settings, to make adjustments like having a voice recording in the computer rather than typing, might be easier*’ (P16).

### Dyslexia informs the career development of dyslexic doctors

#### General practice appeals to dyslexic doctors

Participants often selected general practice as a career owing to its emphasis on interpersonal skills: *‘GP is a natural specialty because we spend a lot of time talking and listening*’ (P3).

Nonetheless, some doctors did choose general practice owing to its shorter training programme and as an alternative choice to hospital medicine:

*‘Surgery is a really practical role and I’m quite practically minded. But I think training is just too long*.’ (P6)

#### Training may not fully prepare dyslexic doctors

Participants commented that 3 years of GP training could be insufficient for dyslexic doctors as training did not necessarily reflect working life post-CCT. During training, the focus was primarily on assessments and time after completion of exams was valuable to learn the ‘*role of a GP*’ (P21). Several participants found training extensions useful:

*‘I had extended training so by the time I got out, I felt more confident. I don't know how someone with three years of training who is dyslexic can work as a GP*.’ (P4)

Furthermore, working full-time could be challenging, with less-than-full-time an option: *‘I went to 60% less-than-full-time because it was getting overwhelming*’ (P12).

Moreover, reasonable adjustments during training did not always follow post-CCT:

*‘GP partners would struggle employing somebody with dyslexia. Potentially you'd have to give more time for admin. Potentially less appointments*.’ (P22)

This was perceived to be related to NHS workforce pressures that were heightened post-CCT:

*‘Realistically, in the current climate and with most practices struggling, they probably won't be able to give that flexibility*.’ (P10)

Nonetheless, educators emphasised that approaching a familiar practice was helpful, in addition to negotiation. However, while some dyslexic GPs successfully negotiated adjustments, this was not always possible:

*‘That hasn't been something that I've successfully been able to negotiate post-training. It’s been, well you can have catch-up slots, but we don't think it’s fair if you have longer appointment times and fewer appointments*.’ (P3)

#### Dyslexic GPs may pursue portfolio careers

Dyslexic GPs could pursue portfolio careers, with locum being one option. Another possibility was working in other clinical environments, such as urgent care or other specialties (for example, emergency medicine, occupational medicine). Other options were non-clinical roles: *‘Some go into research and training. Some have gone into a purely academic side*’ (P14).

#### Non-clinical administration is challenging

Completing non-clinical administration post-CCT was burdensome, including invoices, taxation, and pension forms. Seeking help from accountants was important. Completing annual appraisal and revalidation were difficult, with comparisons drawn to training: *‘I still carry it post-CCT for my appraisal portfolio. I struggle with that*’ (P10).

### Adaptive strategies can enhance performance in GP training assessments

#### Written examinations can be challenging

Participants found written examinations (for example, AKT) difficult with dyslexia, related to issues with *‘reading time, comprehension, and the focus of attention for long periods of time*’ (P1). Moreover, screen-reading could be demanding, in addition to interpretation of some question-stem wording. Several dyslexic participants failed AKT; additional exam time was effective, albeit exhausting.

#### Practical examinations are perceived to be easier

Some participants found practical assessments easier, owing to the focus being on consultation skills: *‘I would outperform in OSCEs* [objective structured clinical examinations] *but underperform in MCQs* [multiple choice questions]’ (P10).

Nonetheless, some participants reported failure (for example, SCA, recorded consultation assessment [RCA]), related to difficulties with reading time, poor verbal expression, reduced interpersonal skills, and time-keeping under pressure. Reasonable adjustments included additional reading time and increased duration of consultations.

#### Workplace-based assessments (WPBAs) can be difficult

Dyslexic residents experienced difficulties with WPBAs. Educators predominantly emphasised issues with dyslexic residents’ portfolio engagement:

*‘They avoid learning logs, and suddenly they're under pressure before an ESR* [educational supervisor report]*. They'll put loads of log entries on, generally not brilliant quality*.’ (P10)

Some educators noted that disorganisation was compounded in IMGs who were *‘completely new to NHS and hit with this IT platform*’ (P26).

Learning log reflections were onerous, owing to challenges with composition and grammar:

*‘I found the portfolio an absolute drain on my life. The learning logs. Oh God, I used to find it so difficult to put things into words*.’ (P3)

Some felt that the portfolio should allow oral entries, while others used artificial intelligence and voice recognition to write reflections:

*‘I would audio-note myself. But if I typed, there would be spelling errors everywhere and it wasn't succinct*.’ (P6)

Some commented that WPBAs requiring organisational efficiency, particularly audits, quality improvement activities, and prescribing assessments were difficult, although others disagreed. Furthermore, for WPBAs involving feedback from supervisors, colleagues, and patients, negative comments occurred, related to residents’ organisation and time-keeping. Other WPBAs, such as case-based discussions, were perceived to be less challenging as others *‘would do most of the writing*’ (P3).

#### Interactive learning is effective

Interactive methodologies were effective for dyslexia, including reading guidelines while seeing patients, note-taking, and annotation. Audiovisual learning was effective, including audio-notes, pictures, tables, podcasts, mind-mapping, mnemonics, and gamification. Study groups, verbal explanations, the Pomodoro technique and question banks were also effective.

Educators emphasised their role. Reviewing mock questions with residents to check clinical reasoning and observing their consultations were valuable. Exam techniques included the cover test, dissecting the question, reviewing question formats and visualisation. Practising time management was important.

### Workplace adjustments can enhance the experiences of dyslexic doctors in general practice

#### Experiences of dyslexia and its strengths are unique to every individual

Participants expounded workplace adjustments to improve processing and time-keeping. Nonetheless, they emphasised that interventions were individualised:

‘O*ne of the dangers with something like dyslexia is saying there is just one way to deal with this because I don't think it’s that homogeneous*.’ (P11)

Participants detailed strengths, including enhanced interpersonal skills, creativity, and lateral thinking: *‘It gives you that ability to connect with people. That’s given me an advantage and is actually an asset to my job*’ (P3).

However, one participant did not feel that dyslexia gave strengths beyond their peers, while another commented that enhanced skills could be diminished by additional burden.

#### Adjustments to GP clinics are helpful

Longer individual appointment times, catch-up slots, and breaks helped improve time-management during clinics:

*‘I have three catch up slots throughout the morning ... My morning finishes later*.’ (P3)

Nonetheless, some commented on the impracticality of lengthy appointment durations for residents: *‘I don't know how much more time we can give. We already give 45 minutes per consultation*’ (P25).

Longer clinics could also be detrimental, including reduced administration time, difficulties undertaking home visits, and being exhausting:

*‘By increasing to 15 minutes for the same amount of patients, it means that I'm constantly consulting for 8 hours straight and mentally I can't do that*.’ (P6)

A further measure was ensuring familiarity, through adequate induction, and maintaining the same clinic room, although this was sometimes unfeasible: *‘It’s got merit in a large organisation, but in a smaller practice you might not have a dedicated room*’ (P22).

Managing patients with complex or multiple issues was challenging and keeping to one issue per appointment could be helpful. Furthermore, documenting information immediately during consultations, could improve time-keeping.

Participants commented on the importance of incorporating adequate supervision for dyslexic residents, with flexibility according to individual needs:

*‘Recognising and working with the trainee. Would they prefer having a debrief after two or three patients, rather than at the end of the surgery?*’ (P16)

#### Administration strategies are helpful

Undertaking administration was time-consuming for dyslexic doctors, including task completion, reviewing letters, processing results, and reviewing and signing prescriptions:

*‘Documents are my worst enemy, especially with the bloods and tasks. I find that very overwhelming*.’ (P6)

Coming in earlier or leaving later was common: *‘Because I came in early, it gave me extra time and space to look through letters slowly and methodically, equally prescriptions as well*’ (P10).

However, other measures included increased scheduled time to undertake administration, and/or a reduced overall number of administrative items, although this raised issues of fairness:

*‘Other trainees get 10 letters per session. I said, let’s try 5 letters instead. He had less administrative tasks to do. But I definitely feel that’s an issue*.’ (P24)

Longer documents were particularly onerous and printing them for annotation was helpful. A further strategy was using the task function to forward tasks to colleagues, and/or to set personal reminders: *‘I just end up with a very big inbox full of stuff to work through. It’s the only way I'm going to remember to do it later*’ (P3).

Minimising interruptions was also important: *‘Having a focused timeframe where you're not going to be interrupted, to sit and go through things, is really helpful*’ (P5).

#### Adaptive technologies are helpful

One adaptive technology was modified headsets that removed *‘distractions*’ (P3). Further technologies including adapted keyboards, speech recognition software, and dictaphones to reduce typing burden. Nonetheless, there were challenges:

*‘They find using dictaphones really difficult because they've still got to formulate what they're saying. They find it easier to just type it*.’ (P15)

Further hardware adaptations included the use of two screens, filters, and modified settings (lighting, colouring, and magnification):

*‘So one thing that was suggested was having two screens. And then my efficiency went through the roof*.’ (P4)

Software technologies included electronic record spellchecker and predictive text. *‘Having a personalised IT setup that works for that individual*’ (P20) was important.

Artificial intelligence for drafting and writing patient notes and referral letters was another measure. However, there were concerns about confidentiality, legal implications, and cost:

*‘So you can use ChatGPT, you speak and it will transcribe for you. But obviously, you've got to be very careful not to have patient identifiable information*.’ (P10)

Participants also used templates or cheat-sheets that could be pasted into notes:

*‘I use templates as much as possible. For children under five, an HRT* [hormone replacement therapy] *one, baby check template*.’ (P3)

#### Team support is important

Dyslexic doctors often struggled to integrate with the GP team as they were *‘always catching up*’ (P8) and because *‘the job is exceptionally isolating. You're on your own for 10 plus hours a day*’ (P6).

Nonetheless, participants did report supportive colleagues and peers that helped navigate through clinics, portfolio, and examinations. In particular, buddy groups and allyships within training and GP networks were beneficial.

Educators emphasised the importance of informing wider practice staff: *‘Remember the wider team, because educators might be involved with supervision, but they might not be formulating the timetable’* (P16).

#### Prescribing strategies are helpful

Prescribing was challenging, with difficulties around drug name similarities and spelling. Strategies included concentration and double-checking:

*‘I'm over cautious. I'll always check the BNF* [British National Formulary]*, check it on EMIS. I don't want to make a mistake because of dyslexia*.’ (P8)

Numerical difficulties included number recall and calculations. Taking time, using a calculator, and breaking information into chunks (‘chunking’) were useful.

Some participants found electronic prescribing helpful, such as predictive text: *‘If you put the first three letters in, it comes up with suggestions. But the problem is if I don't get the first three letters right, like the one that gets me is flucloxacillin*’ (P2).

Another strategy was using electronic drug alerts for interactions, in addition to speaking with colleagues (for example, pharmacist) for complex prescribing.

#### Accessible resources can be helpful

Clinical (for example, National Institute for Health and Care Excellence [NICE]) and training resources (for example, RCGP, deanery) were helpful. Nonetheless, some were less accessible:

*‘If you had dyslexia, trying to understand the information about study leave would be really difficult*.’ (P17)

Resources from third-sector organisations were generic and *‘not really what we have, which is an apprenticeship-type environment*’ (P24).

Therefore, participants felt that a specific *‘toolkit*’ (P23) for GP residents and educators would be useful to *‘help make improvements to identify and support trainees that do have dyslexia*’ (P23).

Participants specified useful content of a toolkit: dyslexia recognition; referral pathways; adaptive strategies for workplace and assessments; and experiences following completion of training. Furthermore, it should be electronic, interactive, and contain stories regarding dyslexic GPs who had completed training. Nonetheless, participants emphasised that toolkits must be used flexibly: *‘So having that ability to see what other people do and the freedom to mix and match*’ (P11).

## Discussion

### Summary

This study has identified five themes exploring dyslexia within general practice. The findings broadly corroborate with experiences in other neurodiversity research.^7,8,10,20,32–38^ However, this study provides novel insights into the lived experiences and strategies of educators, residents, and post-CCT GPs. It highlights particular challenges within general practice, arising from its autonomous nature and workload. The findings are also likely to have relevance to GP training within high-income countries that share similar curricular design (for example, Australia, US)^13^ and workload issues in primary care (for example, the Netherlands, France).^39^

### Strengths and limitations

Measures taken to improve trustworthiness are outlined in the Method section. One particular strength is that 26 participants were included, offering a range of perceptions from residents, GPs, and educators. Themes were broadly identified across all participants although some themes were emphasised within a particular group (see Table 2).

Furthermore, most participants were based in the West Midlands, which may have skewed perceptions. Nonetheless, as GP training requirements are standard across the UK, the findings are likely to be transferrable. Moreover, survivor bias may have influenced participants with an especial interest to partake in the study.

### Comparison with existing literature

One review identified that further research was required regarding dyslexia in GP training, and experiences post-CCT,^8^ which are addressed by this study. As with other articles, the findings highlight the need for inclusive environments for dyslexic doctors. Nonetheless, further training is required for GP educators and residents to improve awareness, identification, and provision of adjustments.^8,20^ This study also suggests that a specific toolkit concerning dyslexia in GP training, which can be utilised by residents and educators, could enhance awareness and knowledge. While a toolkit may improve knowledge and understanding, some level of personal burden may continue even with a supportive environment.

Screening doctors, particularly IMGs, has previously been suggested as a measure to improve identification,^20^ although given concerns by educators in this study regarding overdiagnosis of doctors who will otherwise complete training with no concerns, targeted screening may be appropriate. Nonetheless, one study recommended that all training doctors are screened for dyslexia to improve wellbeing and NHS costs.^40^

Negative reactions, stigma, and lack of support are persistently reported elsewhere.^7–9,20^ Hence, there is a need for a change in attitudes from a deficit model towards valuing neurodiversity. Changing wider societal perceptions, alongside programmatic and individual adjustments, can allow dyslexic GPs and residents to excel, using an interactionist neurodiverse approach.^3^ Peer support, such as networking, allyships, and buddying, can enhance attitudes. This is especially important at the intersection of dyslexia with IMGs, in whom there is existing differential attainment in postgraduate training, notwithstanding dyslexia.^18,20,41^

Moreover, the findings suggest that dyslexic residents may require more than 3 years of training to learn the day-to-day role of a GP. The RCGP First5 scheme can provide mentorship and professional support for independent practice post-CCT for 5 years;^42^ therefore, further exploration is needed regarding support for dyslexic GPs after completion of training and whether existing schemes such as First5 could be sufficient to facilitate this. However, the RCGP has advocated that all training programmes should be extended to 4 years, reflecting the evolving complexity of primary care,^43^ which may be more important for residents with additional needs. Longer training may also align UK general practice with other European nations.^44^

Regarding adaptive strategies, several strategies have been elucidated against each theme, which are summarised in Figure 2, but detailed in greater depth in supplementary table 2. While some adjustments are also reported elsewhere,^8,20^ these are specific to GP settings and should be flexibly applied, according to individual needs and context. Legal provision often means that organisations must offer reasonable adjustments for disabilities, such as the Equality Act 2010^12^ within the UK. Nonetheless, adjustments may not always be considered reasonable, especially where issues of fairness may arise (for example, fewer patients, less administration). Nonetheless, a compromise may be reached such as longer clinics, although this may be associated with more personal burden. Therefore, training organisations and employers must have well-informed policies regarding their provision and undertake discussions with dyslexic residents and GPs.

**Figure 2.**
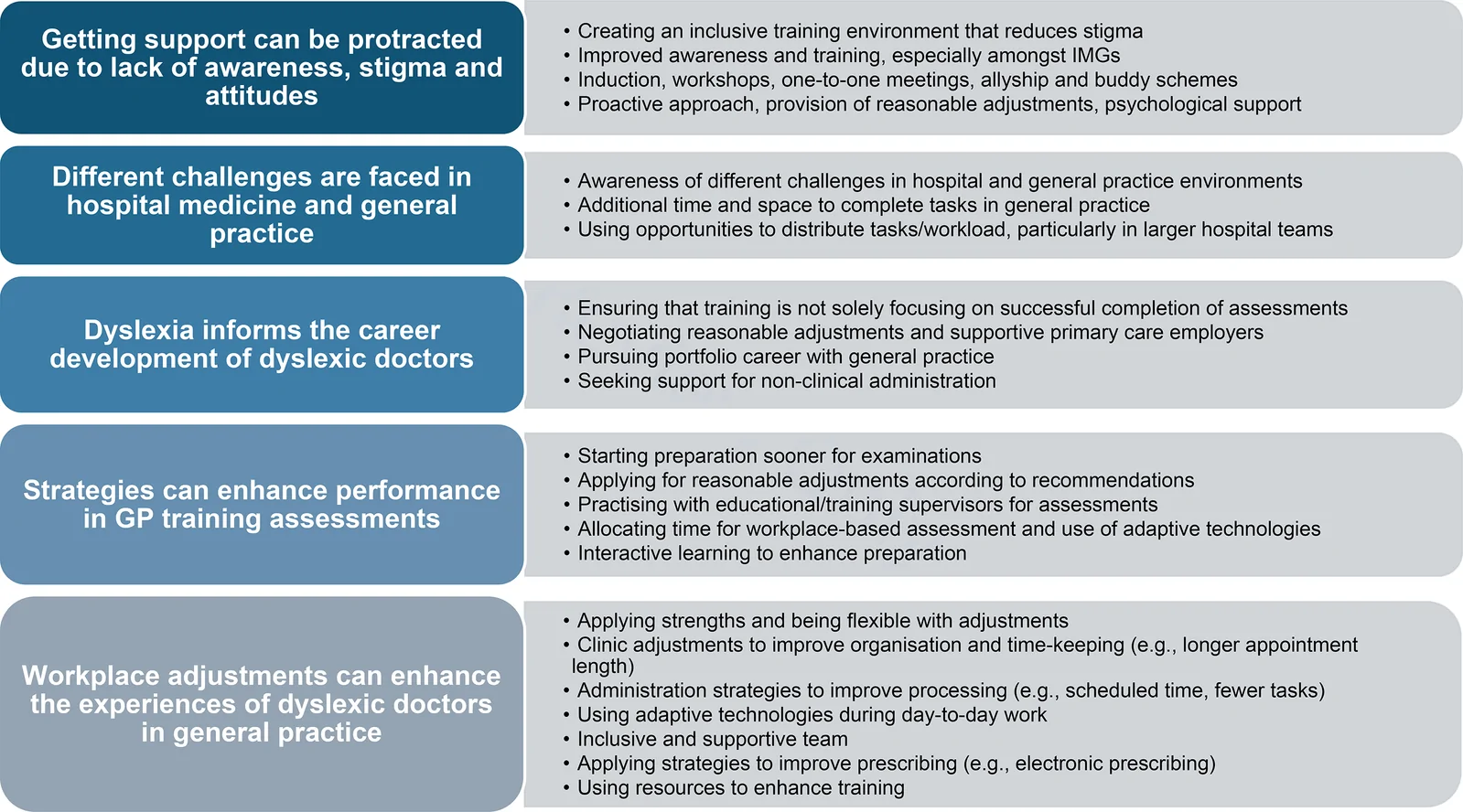
Key adaptive strategies identified, against each theme. IMG = international medical graduate

Moreover, the findings suggest that post-CCT, adjustments are not always provided by employers, reflecting current challenges of NHS workload and access pressures.^39,45,46^ Nonetheless, it indicates a need for inclusivity to permeate beyond GP training and potential strategies post-CCT are outlined in Figure 2 and supplementary table 2. Measures to prioritise support and wellbeing may improve workforce retention.^47^

All assessment components — the AKT, SCA, and WPBAs — can be challenging with dyslexia, also identified in other studies.^8,20^ The RCGP recently updated the AKT format, which may benefit candidates with SpLD or dyslexia.^48^ The SCA, introduced in 2023, replaced the RCA and CSA, which were both associated with differential attainment for dyslexic candidates.^15,16^ There is a current lack of published studies regarding SCA performance and SpLD or dyslexia.

While WPBAs are broadly viewed positively by educators and residents,^49^ the findings suggest that dyslexic residents struggle with those involving written communication, organisation, and feedback, leading to adverse annual training review outcomes.^15,18^ Nonetheless, WPBA strategies can be implemented to mitigate some of these issues (supplementary table 2).

### Implications for research and practice

Further training is required regarding identification and adaptive strategies for dyslexia, for educators, residents, and employers, including post-CCT. Furthermore, training programmes and employers should foster a positive culture to improve inclusivity, through improved awareness, promoting disclosure, and offering and implementing reasonable adjustments. Differences in the challenges for dyslexic doctors within hospital medicine and general practice should be acknowledged and reflected in the adaptive strategies provided within primary care. Moreover, creating a GP-specific toolkit could support improved experiences of dyslexic doctors in GP training. Further research is required on dyslexia among IMG doctors, and on the impact of recent changes with UK-based examinations (AKT and SCA).

## Data

The dataset relied on in this article is available from the corresponding author on reasonable request.
